# Fast Profiling of Natural Pigments in Different *Spirulina (Arthrospira platensis)* Dietary Supplements by DI-FT-ICR and Evaluation of their Antioxidant Potential by Pre-Column DPPH-UHPLC Assay

**DOI:** 10.3390/molecules23051152

**Published:** 2018-05-11

**Authors:** Eduardo Sommella, Giulio Maria Conte, Emanuela Salviati, Giacomo Pepe, Alessia Bertamino, Carmine Ostacolo, Francesca Sansone, Francesco Del Prete, Rita Patrizia Aquino, Pietro Campiglia

**Affiliations:** 1 Department of Pharmacy, University of Salerno, Via Giovanni Paolo II 132, I-84084 Fisciano, SA, Italy; esommella@unisa.it (E.S.); gconte@unisa.it (G.M.C.); esalviati@unisa.it (E.S.); gipepe@unisa.it (G.P.); abertamino@unisa.it (A.B.); fsansone@unisa.it (F.S.); aquinorp@unisa.it (R.P.A.); 2 PhD Program in Drug Discovery and Development, University of Salerno, Via Giovanni Paolo II 132, I-84084 Fisciano, SA, Italy; 3 Department of Pharmacy, University of Naples Federico II, Via D. Montesano 49, I-80131 Napoli, Italy; ostacolo@unina.it; 4 Department of Biology, University of Naples Federico II, Via Mezzocannone 16, I-80131 Napoli, Italy; francesco.delprete@unina.it; 5 European Biomedical Research Institute of Salerno, Via De Renzi 50, I-84125 Salerno, Italy

**Keywords:** carotenoids, DIMS, DPPH, FT-ICR, Spirulina, UHPLC

## Abstract

*Arthrospira platensis*, better known as Spirulina, is one of the most important microalgae species. This cyanobacterium possesses a rich metabolite pattern, including high amounts of natural pigments. In this study, we applied a combined strategy based on Fourier Transform Ion Cyclotron Resonance Mass Spectrometry (FT-ICR-MS) and Ultra High-Performance Liquid Chromatography (UHPLC) for the qualitative/quantitative characterization of Spirulina pigments in three different commercial dietary supplements. FT-ICR was employed to elucidate the qualitative profile of Spirulina pigments, in both direct infusion mode (DIMS) and coupled to UHPLC. DIMS showed to be a very fast (4 min) and accurate (mass accuracy ≤ 0.01 ppm) tool. 51 pigments were tentatively identified. The profile revealed different classes, such as carotenes, xanthophylls and chlorophylls. Moreover, the antioxidant evaluation of the major compounds was assessed by pre-column reaction with the DPPH radical followed by fast UHPLC-PDA separation, highlighting the contribution of single analytes to the antioxidant potential of the entire pigment fraction. β-carotene, diadinoxanthin and diatoxanthin showed the highest scavenging activity. The method took 40 min per sample, comprising reaction. This strategy could represent a valid tool for the fast and comprehensive characterization of Spirulina pigments in dietary supplements, as well as in other microalgae-based products.

## 1. Introduction

In the last decade, the food and pharmaceutical sector has been driven towards an interest in natural compounds that, if assumed on a daily basis, could bring benefits to human health, especially in the treatment of chronic diseases [1]. This led to the development of so-called nutraceuticals and functional foods, whose market is continually growing, helped by the interest of healthy conscious consumers. Among the natural matrices rich in bioactive compounds, microalgae represent one of the most promising [2]. These microorganisms are a source of various biologically active molecules, including aminoacids, polyunsaturated fatty acids, minerals, proteins and pigments [3]. Additionally, they are capable of growing in different aquatic environments, and can tolerate different temperatures, pH and salinities [4]. *Arthrospira platensis*, also known as Spirulina, is one of the most economically important species. This blue-green microalga has been widely commercialized and is sold in different forms, including as powder, tablets and creams. The high interest in Spirulina derives from numerous healthy properties attributed to the consumption of this microalga, which include antihypertensive, hypolipidemic, anticancer and antioxidant properties [5]. These properties are related to the outstanding content of biocompounds; in particular, Spirulina is characterized by a protein content that is roughly ten times higher than that of soybean [6]; moreover, it contains essential amino acids, polyunsaturated fatty acids, vitamins and pigments. Spirulina-based products are employed by athletes as anti-fatigue and amino acid supply, and for their anti-aging detoxifying and antioxidant properties in cosmetics. The antioxidant potential of Spirulina is partially attributed to the high content of natural pigments, especially carotenoids, which are also recognized as having numerous healthy benefits [7]. Carotenoids are a group of molecules characterized by a C40 chain of isoprene units synthetized by plants and microorganisms, and are usually colored [8]. They are distinct in primary, which are involved in the photosynthetic apparatus, and in secondary, that are produced by microalgae following a particular situation of stress [9]. Despite the success of Spirulina in the market, the profiling of pigment in this species has been only partially described. The determination of carotenoids in Spirulina has been carried out mainly by liquid chromatography (LC) coupled with diode array (DAD) and mass spectrometry (MS) detection by employing low-resolution mass analyzers [10], and recently also by high-performance thin layer chromatography (HPTLC) [11]. Given that the Spirulina pigment fraction is highly complex, conventional LC-MS-based methods suffer from a low separation efficiency, long analysis time, and low mass accuracy, which can result in inaccurate identification. Furthermore, the antioxidant activity of carotenoid extracts has been evaluated by spectrophotometric or enzymatic assays [12,13], which reflect only the total activity of the extract, without adequate measure of the antioxidant potential of individual molecules. Due to the increasing commercial interest in Spirulina, faster and more highly efficient analytical tools are required to characterize the final products and raw materials. In this regard, the objective of this study was to develop a combined platform for the qualitative and quantitative characterization of Spirulina pigments in different dietary supplements. To tackle such a task, we exploited the accurate mass measurement and resolution of Fourier Transform Ion Cyclotron (FT-ICR) for the qualitative profiling of the extract in both direct infusion (DIMS) mode or coupled with Ultra High-Performance Liquid Chromatography (UHPLC). Moreover, the antioxidant potential of major carotenoids was evaluated by the combination of pre-column reaction with 2,2-diphenyl-1-picrylhydrazyl radical (DPPH) followed by UHPLC separation, in order to obtain information regarding the contribution of individual pigments to the global antioxidant activity.

## 2. Results and Discussion

### 2.1. High-Resolution Mass Spectrometry Profiling of Spirulina Pigments

**Figure 1:**
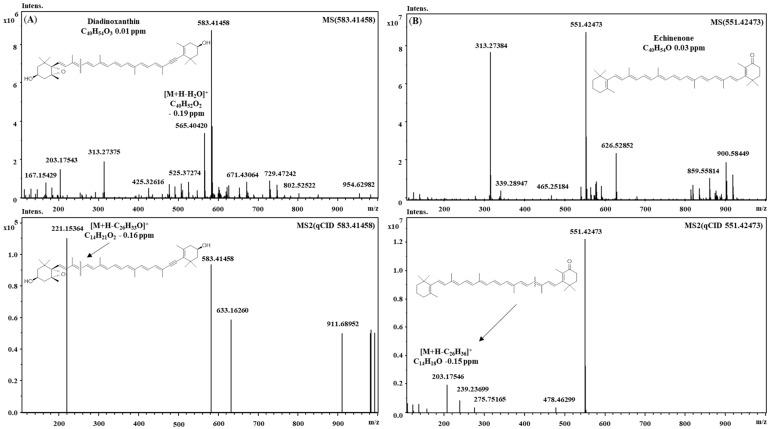
MS (**top**) and MS/MS (**bottom**) spectra showing structure elucidation and fragmentation pattern of peak 3 diadinoxanthin (**A**) and peak 18 echinenone (**B**).

**Figure 2:**
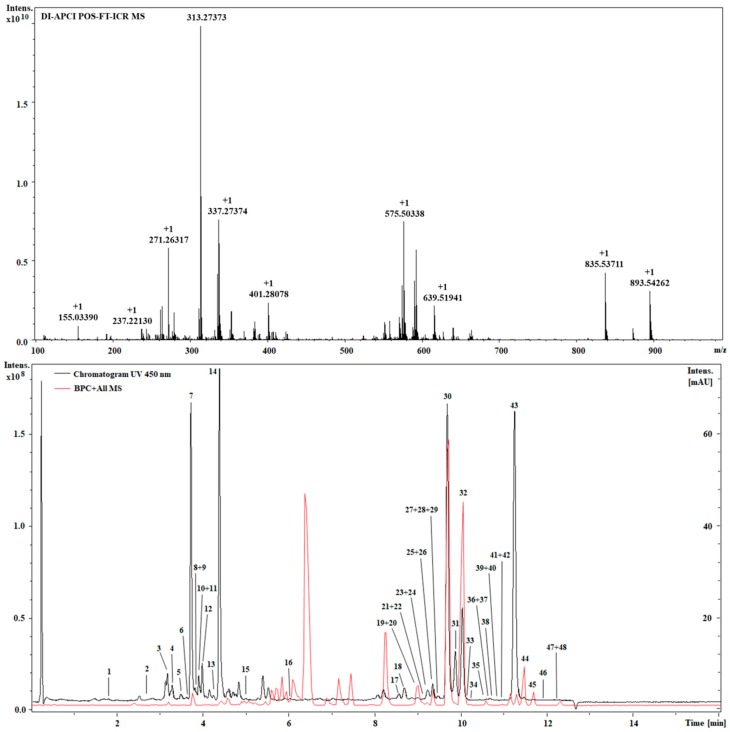
DIMS-APCI (**top**) and LC-APCI-FT-ICR MS (**bottom**) identification of Spirulina pigments.

FT-ICR-MS is characterized by unmatched ultra-high mass accuracy and resolution, which are ideal for the analysis of complex phytochemical samples [14]. In this approach, we employed APCI ionization, which outperformed electrospray for almost all analytes in both DIMS and LC-MS modes (data not shown). Table 1 shows the tentative identification relative to both approaches. As can be observed, a higher number of tentatively identified compounds (51) was obtained by both DIMS and LCMS with respect to previous investigations on Spirulina [10]. Ultra-high mass accuracy was obtained for DIMS (≤0.01 ppm), while slightly higher values were obtained for LC-MS. This is relative to the intrinsic nature of FT-ICR, since the long scanning time required for ultra-high resolution is not very compatible with fast UHPLC timescales [15]. Contrariwise, direct infusion, which introduces constant ion flow, makes it possible to obtain the highest sensitivity, as well as accuracy and resolution. Ultra-high mass accuracy is highly beneficial to unambiguous formula assignment, and compounds can be identified often only by accurate mass [16]. Different carotenoid classes were present: hydroxyl, epoxy and ketocarotenoids, as well as carotenes; several compounds are reported for the first time in Spirulina (Table 1). Among hydroxycarotenoids, the peak at rt: 3.69 possessing strong absorbance at 450 nm, showed the typical loss of water [M + H − 18]^+^, with a molecular formula of C_40_H_54_O_2_, and thus it was tentatively proposed to be diatoxanthin. Regarding epoxy derivatives, the peak at rt: 3.29 was characterized by the diagnostic fragment ion at *m*/*z* 221 (C_14_H_21_O_2_), which is derived from the cleavage between the C10 and C11 bond (Figure 1A) and was tentatively identified as diadinoxanthin. Similarly, other compounds of this class showed an analogous fragmentation pattern [17]. Compound 18 exhibited a diagnostic fragment ion at 203 *m*/*z* (C_14_H_18_O), which points out a keto group on the B-ring. This fragment is observed in keto derivatives [18] deriving from the fragmentation at C10–C11, in which the positive charge is retained on the ketone moiety. Thus, this information and the molecular formula C_40_H_54_O, leads to possible identification as echinenone (Figure 1B). A large number of chlorophyll derivatives were detected, and some of them, such as divinyl chlorophyll a, presented the fragment at *m*/*z* 614 (C_35_H_34_MgN_4_O_5_), which indicates a phytyl chain loss. Hydroxylated derivatives of pheophytin and chlorophyll a showed a mass difference of 16 Da with respect to accurate mass, and their fragmentation patterns showed a loss of water [−18 Da]. Hydroxylated compounds could derive from transformations that occur during the extraction process [19]. A further benefit of DIMS is the analysis time, which was half of the LCMS method: 4 min vs. 16 min (Figure 2). Clearly, one of the drawbacks of DIMS is the inability to separate isomers, which, on the other hand, is possible using chromatography. The DIMS approach is highly useful when combined with other complementary techniques, as showed recently by several authors [20].

### 2.2. Quantitative Profile of Spirulina Pigments by UHPLC-PDA

Separation of natural pigments is usually performed by C18 and C30 columns [21], the latter are usually best suited for the separation of geometric isomers. Numerous evidence indicates the benefits of using sub-3 and sub-2 micrometers particles, either fully (FPP) or superficially porous (SPP), in the analysis of natural compounds [22,23]. This has also been recently shown for carotenoids in UHPLC conditions [24], as well as SFC [25]. In this work, we employed and compared both 1.7 µm FPP and SPP C18 and 2.6 µm SPP C30 columns. As can be seen from Figure S1, separation on the three columns were similar, except that a better separation of alfa, *trans* and *cis* beta carotene was obtained on the C30. The employed column (namely Accucore^TM^ C30) is the only sub-3 micrometer C30 column on the market. The stationary phase is endcapped, which, in comparison to non-endcapped polymeric C30 phases, provides no appreciable separation of isomers [21]. The employed gradients were optimized for instrument and column characteristics. The choice was made in order to obtain acceptable separations in less than 15 min analysis time; in this regard, it should be pointed out that the non-endcapped C30 columns employed in numerous papers are characterized by very long analysis times [26], which were unpractical for the scope of this study. Due to the high cost of standards, only major carotenoids were quantified, quantification was performed by PDA at the maximum of absorbance. The objective of this study was to highlight the profiles and differences among two commercial and one lab-made Spriulina-based products. Table 2 shows that the most abundant compounds were diatoxanthin (363.96 ± 1.03), zeaxanthin (362.51 ± 0.51) and beta carotene (2388.83 ± 14.94). Among the three formulations, both powders showed in total higher amounts of pigments with respect to tablets (1637.86 ± 8.41 μg/g and 2551.49 ± 6.86 μg/g, respectively vs. 1384.18 ± 7.55 μg/g). This aspect could be due to the different technological processes, during the production of final products. It is noteworthy that chlorophylls were highly abundant, but these were not quantified due to lack of standards. The employed method was able to resolve zeaxanthin from lutein, which are usually difficult to separate, and thus quantify, revealing that the latter was not present in the sample, as shown by the overlapped standards chromatogram in Figure S2. By taking advantage of the low dwell volume of the UHPLC system employed, the analysis time was kept under 15 min (10 min for the C18). Repeatability was established by triplicate injections of sample and solutions at low, medium, and high concentration levels of the calibration curve on the same day, and within two consecutive days; limits of detection (LODs) and quantification (LOQs) were calculated by the ratio between the standard deviation (SD) and analytical curve slope multiplied by 3 and 10, respectively. Results are reported in supplementary Tables S1 and S2.

### 2.3. Evaluation of Antioxidant Potential of Single Pigments by DPPH-UHPLC-PDA

**Figure 3:**
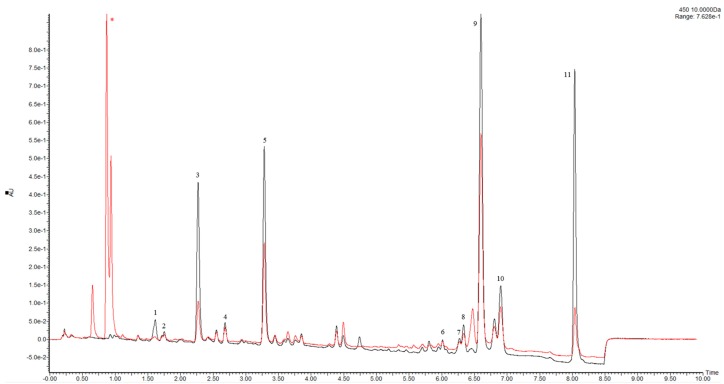
UV/Vis-UHPLC- (450 nm) chromatograms of Spirulina pigment extract before (black) and after (red) reaction with DPPH radical (marked with an asterisk).

The DPPH free radical assay is well known as an easy and rapid way to determine antioxidant activity, and is widely used for natural and food samples. Regarding complex multianalyte samples, one of its drawbacks is its inability to provide information regarding the individual antioxidant potential of analytes. Contrariwise, if the assay is coupled with a separation technique, such as UHPLC, the method can be useful for the screening of individual antioxidants [27]. In this case, after the reaction with the radical, the peak areas (UV/Vis) of potential antioxidants would decrease [28]. In this study, a pre-column reaction with DPPH radical was performed prior UHPLC separation. Two crucial aspects are: the ratio between the concentration of DPPH and the extract, and the reaction time. If an excess of DPPH is employed, the differences in the antioxidant activity cannot be measured, since every peak just disappears into the UV/Vis trace; on the contrary, with an inadequate concentration of DPPH, no significant differences can be observed. After several tests, we found that the best conditions were obtained with 0.5–0.9 mM of DPPH. The optimal reaction time was 30 min, which was determined by injecting at different time intervals; after this point, no further changes in peak areas were observed, with the color of the solution being stably yellow. The UV/Vis chromatogram (450 nm) related to the separation of both untreated and DPPH-spiked Spirulina sample is shown in Figure 3. As can be appreciated, several peaks were significantly reduced, while others remained almost unchanged. In particular, among the pigments in Table 2, as expected, β-carotene showed higher scavenging activity with respect to xanthophylls such as zeaxanthin and antheraxanthin, which is known to be related to the presence of hydroxyl substituents on the B-ring [29]. Interestingly, diadinoxanthin and diatoxanthin possessed a higher scavenging activity with respect to other xanthophylls. This difference is probably due to the triple bond, which is known to increase the oxidation potential, as previously reported [30,31]. These aspects have not been reported so far, and require further investigation with other antioxidant assays, such as ABTS. The comparison of the IC_50_ of the three dietary supplements revealed that the formulation with the lowest IC_50_ is the lab-made Spirulina powder (1.21 mg/mL), whereas the other dietary supplements possess similar values (2.99 mg/mL vs. 2.68 mg/mL). This is clearly related to the highest amount of pigment being contained in the lab-made powder (b), whereas the high content of chlorophylls, which are known to be antioxidant compounds [32], in the powder (a) with respect to tablets results in a lower IC_50_. The DPPH-UHPLC method took only 40 min per sample comprising reaction. In comparison to online methods, which require an additional pumping system and a reactor coil, the employed setup is easier and can take full advantage of the higher efficiency and speed of UHPLC [27], not being affected by the extra-column contributions.

## 3. Material and Methods

### 3.1. Chemicals

Ultra-pure water (H_2_O) was obtained by a Direct-8 Milli-Q system (Millipore, Milan, Italy); LC-MS grade acetonitrile (ACN), methanol (CH_3_OH), 2-propanol (IPA), water (H_2_O), ammonium acetate (CH_3_COONH_4_), and standards of β-carotene, lutein, and zeaxanthin, were all purchased from Sigma-Aldrich (St. Louis, MO, USA). Spirulina powders and tablets were respectively purchased from FarmaLabor SRL (Canosa di Puglia, Barletta-Trani, Italy) and Dr. Giorgini (Bologna, Italy). Lab-made Spirulina powder was kindly donated by a local farmer.

### 3.2. Sample Extraction

Pigment extraction was carried out as follows. 350 mg of Spirulina powder (tablets were prior pulverized in a mortar) were treated with 50 mL of ethanol fortified with 20 µg/mL of BHT. The sample was subjected to 15 min of ultrasound (550 W of power), then the suspension was stirred for 30 min at room temperature and then centrifuged (10 min × 6000 rpm at 25 °C). At the end, the supernatant was removed, and the pellet was retreated following the same protocol another four times. Finally, the supernatants were pooled and lyophilized. The same conditions were employed for each sample of Spirulina.

### 3.3. Instrumentation

High-resolution MS analyses were performed on a SolariX XR equipped with a 7T magnet (Bruker Daltonics, Bremen, Germany). An APCI source operating in positive ionization was used. For direct infusion mode, samples were injected with instrument syringe pump (250 µL), while for LC-HRMS, an Ultimate 3000 UHPLC (Thermo Scientific, Bremen, Germany) system was employed. DPPH-UHPLC-PDA analyses were performed on an Acquity *I* class, equipped with a QDa mass detector (Waters, Milford, MA, USA) system.

### 3.4. Columns

A Waters^®^ Acquity UPLC^®^ BEH C18 50 mm × 2.1 mm, 1.7 µm was employed for all analyses. A Thermo Accucore^TM^ C30 100 mm × 2.1 mm, 2.6 µm and a Phenomenex^®^ (Castelmaggiore, Bologna, Italy) Kinetex^®^ EVO C18 100 mm × 2.1 mm, 1.7 µm, were employed for purposes of comparison.

### 3.5. DIMS and LC-HRMS Parameters

The instrument was tuned with a standard solution of sodium trifluoracetate (NaTFA). Samples (10 µg/mL in CH_3_OH) were infused at 50 µL/min. Mass Spectra were recorded in broadband mode in the range 150–3000 *m*/*z*, with an ion accumulation of 20 ms, with 200 scans using 8 million data points (8M). Nebulizing (N_2_) and drying gases (air) were set at 1 and 4 mL/min, respectively, with a drying temperature of 200 °C. MS/MS of the ion of interest was obtained by isolation in the quadrupole and ramping the collision energy manually. LC-HRMS analyses were carried out with the same parameters, with the exception of: 2 million data points were used (2M), ion accumulation was 80 ms, nebulizing and drying gases were 2 and 8 mL/min, and drying temperature was 250 °C. MS/MS was performed in data-dependent mode, and dynamic collision energy ramp was used. Compound: 2,2,4,4,6,6-Hexakis(2,2-difluoroethoxy)-1,3,5-triazatriphosphinine (*m*/*z* 622.028960) (Apollo Scientific, Bredbury, UK) was employed as lock mass for LC-MS/MS. Mobile phases were: (A) 10 mM CH_3_COONH_4_ in H_2_O *v*/*v*; (B) ACN/CH_3_OH/IPA 70/20/10, flow rate was 0.4 mL/min, LC gradient was: 0 min, 40%B, 6.25 min, 85%B, 9.50 min, 92%B, 10 min, 98%B hold for 1 min, column oven was set at 45 °C, 2 μL of sample was injected. The instrument was controlled by Bruker FTMS Control, MS spectra were elaborated with Compass Data Analysis version 4.2 (Bruker Daltonics, Bremen, Germany), identification of compounds based on accurate MS and MS/MS measurements was performed by Compound Crawler ver. 3.0 (Bruker).

### 3.6. DPPH-UHPLC-PDA Parameters

The determination of antioxidant activity was carried out as previously developed [27]. One hundred microliters of ethanolic extract of Spirulina (2 mg/mL) and 100 μL of DPPH solution (ranging from 0.5 to 0.9 mM) in a 1:1 ratio were briefly mixed and allowed to react for 30 min in the dark at room temperature. 100 μL of methanol was added to the Spirulina extract as control. After filtration with a 0.45-μm filter, 2 μL of sample solution were injected for UHPLC analysis with the following conditions: mobile phases were the same as in Section 3.5, while the gradient was tuned for the different LC instrument: 0.01 min 60%B, 0.75 min 75%B, 3.25 min 85%B, 5.00 min 95%B, hold for 2 min, 7.01 min 100%B, hold for 1 min. Flow rate was 0.4 mL/min. Column oven temperature was set to 45 °C. PDA sampling rate was 20 Hz, time constant 80 ms, data acquisition was set in the range 190–800 nm, and chromatograms were monitored at 450 nm. QDa mass analyzer was operated under Selected Ion Monitoring (SIM) in ESI positive ionization, by specifying the *m*/*z* values of compounds detected with the HRMS approach. MassLynx 4.0 (Waters, Milford, MA, USA) was employed for data analysis.

The change in the pigments’ peak areas of analytes (*PA_control_*) between control and DPPH-spiked (*PA_spiked_*) sample was used to evaluate the antioxidant power according to the following equation:

(1) $$\mathrm{Radical}\;\mathrm{scavenging}\;=\frac{PA_{control}-PA_{spiked}}{PA_{control}}\times 100$$

where (*PA_control_*) refers to the Spirulina extract diluted with methanol, whereas (*PA_spiked_*) refers to the DPPH solution mixed with the Spirulina extract. The percentage of DPPH scavenging versus the concentration of samples was plotted. Whereas, by monitoring the DPPH peak at 517 nm, the concentration of ethanolic extract necessary to decrease the DPPH concentration by 50% was obtained by interpolation from a linear regression analysis and denoted as the IC_50_ value (μg/mL). All determinations were performed in triplicate. 

### 3.7. Qualitative and Quantitative Analysis

Since standards were not available for all compounds, zeaxanthin, lutein and β-carotene were selected as external standards for the quantification. Stock solutions (1 mg/mL) were prepared in methanol/MTBE (70:30) and the calibration curves were obtained in a concentration range, respectively, of 0.25–25 μg/mL (R^2^ = 0.999), 1–100 μg/mL (R^2^ = 0.996), and 1–100 μg/mL (R^2^ = 0.996), with seven concentration levels, and triplicate injections of each level were run. Peak areas were plotted against corresponding concentrations. The amount of the compounds in the sample was expressed as micrograms per gram. Xanthophylls were quantified as zeaxanthin equivalents.

## 4. Conclusions

The developed analytical strategy, consisting of FT-ICR and UHPLC-PDA, provided a detailed definition of the Spirulina pigment fraction. DIMS-FT-ICR, thanks to its ultra-high resolution and mass accuracy, is a promising tool for in-depth profiling of microalgae pigments. The DPPH-UHPLC-PDA method revealed that two xanthophylls, namely diadinoxanthin and diatoxanthin, possess relevant radical scavenging activity. This study further confirms the content of high-value biocompounds in Spirulina, and its importance for the nutraceutical and pharmaceutical field.

## Figures and Tables

**Table 1:** DIMS-APCI and LC-APCI-FT-ICR MS identification of Spirulina pigments.

Peak	rt	Compound	Molecular Formula	[M + H]^+^ DIMS-APCI	Error ppm DIMS-APCI	[M + H]^+^ LC-APCI-FT-ICR MS	MS/MS	Error ppm LC-APCI-FT-ICR MS
1	1.87	Apo-12-Violaxanthal ^1^	C_25_H_34_O_3_	383.25809	−0.05	383.25815	365.24754 ^2^, 347.23705 ^3^	−0.21
2	2.76	Vaucheriaxanthin ^1^	C_40_H_56_O_5_	617.42010	−0.08	617.41998	599.40933 ^2^, 581.39890 ^3^	0.12
3	3.29	Diadinoxanthin ^1^	C_40_H_54_O_3_	583.41458	−0.02	583.41456	565.40420 ^2^, 221.15364	0.01
4	3.33	Canthaxanthin	C_40_H_52_O_2_	565.40402	−0.01	565.40404	547.39350 ^2^	−0.06
5	3.45	Ethyl β-apo-8′-carotenoate ^1^	C_32_H_44_O_2_	461.34143	−0.04	461.34155		−0.32
6	3.67	Adonirubin ^1^	C_40_H_52_O_3_	581.39892	0.01	581.39891		0.02
7	3.69	Diatoxanthin ^1^	C_40_H_54_O_2_	567.41967	−0.02	567.41971	221.13248, 549.40979 ^2^	−0.09
8	3.78	β-Apo-8′-carotenal ^1^	C_30_H_40_O	417.3152	−0.01	417.31542	399.30463 ^2^, 293.22642	−0.34
9	3.85	Hexadehydro-β,β-caroten-3-ol ^1^	C_40_H_50_O	547.39347	−0.06	547.39344		−0.36
10	3.88	Rhodoxanthin ^1^	C_40_H_50_O_2_	563.38838	−0.04	563.38856	545.37778 ^2^	−0.36
11	3.90	Astaxanthin	C_40_H_52_O_4_	597.39382	0.02	597.39384		0.01
12	4.04	Antheraxanthin ^1^	C_40_H_56_O_3_	585.43023	−0.02	585.43029	567.41961 ^2^, 549.40920 ^3^, 493.40407	−0.12
13	4.20	Myxoxanthophyll	C_46_H_66_O_7_	731.48807	0.08	731.48917		−1.42
14	4.38	Zeaxanthin	C_40_H_56_O_2_	569.43529	0.02	569.43552	551.42497 ^2^, 459.36256	−0.37
15	5.06	10-Apo-β-carotenal ^1^	C_27_H_36_O	377.28389	−0.06	377.28403		−0.37
16	6.10	α-tocopherol ^1^	C_29_H_50_O_2_	431.38835	0.01	431.37971		−0.91
17	8.73	Chlorophyll *a* isomer	C_55_H_72_MgN_4_O_5_	893.54226	−0.03	893.54274	555.22547, 481.18779, 614.23848	−0.17
18	8.74	Echinenone ^1^	C_40_H_54_O	551.42473	0.02	551.42473	203.17531	0.03
19	9.00	Pyrochlorophyll *b* ^1^	C_53_H_68_MgN_4_O_4_	849.51640	−0.03	849.51620		0.21
20	9.00	Pheophytin *a* derivate ^1^	C_55_H_72_N_4_O_5_	869.55755	0.01	869.55795		−0.13
21	9.10	Chlorophyllide *b* ^1^	C_35_H_32_MgN_4_O_6_	629.24050	0.01	629.22498		−0.76
22	9.12	Chlorophyll *b* ^1^	C_55_H_70_MgN_4_O_6_	907.55824	0.01	907.55841		−0.19
23	9.15	Pyrochlorophyll *a* ^1^	C_53_H_70_MgN_4_O_3_	835.53711	0.01	835.53746		−0.42
24	9.18	Pyrochlorophyllide *a* ^1^	C_33_H_32_MgN_4_O_3_	557.23978	−0.04	557.23992		−0.29
25	9.26	Pyrochlorophyllide *b*	C_33_H_30_MgN_4_O_4_	571.21901	0.02	571.21902		0.01
26	9.28	OH-Chlorophyll *a* ^1^	C_55_H_72_MgN_4_O_6_	909.53746	0.05	909.53786	525.21366, 553.20861	−0.95
27	9.32	Protochlorophyllide *a* ^1^	C_35_H_32_MgN_4_O_5_	613.22959	−0.01	613.22998		−0.64
28	9.32	13-OH-Chlorophyllide *a* ^1^	C_35_H_34_MgN_4_O_6_	631.24015	0.01	631.24054		−0.62
29	9.32	Divinyl Chlorophyll *a* ^1^	C_55_H_70_MgN_4_O_5_	891.52691	0.04	891.52705	555.22506, 614.23423	0.21
30	9.56	Chlorophyll *a*	C_55_H_72_MgN_4_O_5_	893.54262	−0.03	893.54274	555.22547, 481.18779, 614.23848	−0.17
31	10.03	Cryptoxanthin ^1^	C_40_H_56_O	553.44040	0.01	553.44047	535.430052, 461.37769	−0.15
32	10.04	Chlorophyll *a* isomer	C_55_H_72_MgN_4_O_5_	893.54262	−0.03	893.54274	555.22547, 481.18779, 614.23848	−0.17
33	10.10	Chlorophyllide *a* ^1^	C_35_H_34_MgN_4_O_5_	615.24526	−0.04	615.2461		−0.34
34	10.23	Pheophytin *b* ^1^	C_55_H_72_N_4_O_6_	885.55233	0.14	885.53330		−0.09
35	10.30	15-OH-Lactone-Chlorophyll *a* ^1^	C_55_H_73_MgN_4_O_7_	925.53199	0.47	925.53324		0.89
36	10.47	Pyropheophorbide *b* ^1^	C_33_H_32_N_4_O_4_	549.24967	−0.08	549.24980		−0.31
37	10.48	15-OH-Lactone-Pheophytin *a* ^1^	C_55_H_73_N_4_O_7_	903.56328	−0.28	903.56341	537.24965, 547.23401, 607.25553	−0.09
38	10.79	Chlorobactene ^1^	C_40_H_52_	533.41416	0.03	533.41406		0.21
39	11.03	Chlorophyll *a* derivate I ^1^	C_55_H_68_MgN_4_O_5_	889.51122	0.08	889.51165		−0.41
40	11.06	Phytoene ^1^	C_40_H_64_	545.50810	−0.03	545.50829		−0.39
41	11.12	13-OH-Pheophorbide *a* ^1^	C_35_H_36_N_4_O_6_	609.27078	−0.02	609.27091		−0.24
42	11.12	OH-Pheophytin *a*	C_55_H_73_N_4_O_6_	887.56810	0.01	887.56826	531.23918, 559.23402, 591.26022	−0.17
43	11.16	β-carotene	C_40_H_56_	537.44547	0.01	537.44562	413.32058, 445.38298	−0.27
44	11.21	Octadehydro-β,β-carotene ^1^	C_40_H_48_	529.38288	0.03	529.38303		−0.29
45	11.41	Pheophytin *a*	C_55_H_74_N_4_O_5_	871.57318	0.02	871.57254	593.27615, 533.25473, 519.23921	0.75
46	11.68	Pheophorbide *a* ^1^	C_35_H_36_N_4_O_5_	593.27583	0.02	593.27601		−0.27
47	12.30	Pyropheophorbide *a* ^1^	C_33_H_34_N_4_O_3_	535.27037	0.01	535.27058		0.39
48	12.30	Pyropheophytin *a* ^1^	C_53_H_72_N_4_O_3_	813.56769	0.04	813.56787	535.27058, 507.27549, 461.23369	−0.18
49		δ-tocopherol ^1^	C_27_H_46_O_2_	403.35706	0.01			
50		γ-tocopherol ^1^	C_28_H_48_O_2_	417.37270	0.02			
51		Phytofluene ^1^	C_40_H_62_	543.49242	0.01			

^1^ Detected for the first time in *Spirulina (Arthrospira platensis)*; ^2^ [M + H − H_2_O]^+^; ^3^ [M + H − H_2_O − H_2_O]^+^.

**Table 2:** Quantitative data, RSA% of single compounds and IC_50_ of different dietary supplements.

		Dietary Supplement Powder	Lab Made Powder	Dietary Supplement Tablet		
		Quantitative				
Peak	Compounds	μg/g	μg/g	μg/g	RSA %	
1	**Diadinoxanthin**	28.01 ± 0.11	55.27 ± 0.16	30.79 ± 0.05	15.07 ± 0.17	
2	**Alloxanthin/Canthaxanthin**	22.76 ± 0.04	26.38 ± 0.17	25.79 ± 0.03	6.66 ± 0.27	
3	**Diatoxanthin**	100.11 ± 0.22	363.96 ± 1.03	78.33 ± 0.29	14.45 ± 0.23	
4	**Antheraxanthin**	27.20 ± 0.02	31.60 ± 0.15	28.45 ± 0.11	5.99 ± 0.11	
5	**Zeaxanthin**	113.76 ± 0.15	362.51 ± 0.61	91.95 ± 0.32	10.02 ± 0.05	
6	**Echinenone**	24.95 ± 0.16	25.05 ± 0.09	32.57 ± 0.15	4.54 ± 0.15	
7	**β-carotene**	1226.99 ± 7.67	1544.36 ± 4.06	988.47 ± 6.10	16.23 ± 0.30	
		**Dietary Supplement Powder (a)**	**Lab Made Powder (b)**	**Dietary Supplement Tablets**	**Ascorbic Acid**	**BHT**
**IC_50_ (mg/mL)**		2.99 ± 0.05	1.21 ± 0.02	2.68 ± 0.03	0.03 ± 0.002	0.02 ± 0.001

## Supplementary Materials

The Supplementary Materials are available at http://www.mdpi.com/1420-3049/23/5/1152/s1.
