# Supplementary material for: Fast Profiling of Natural Pigments in Different Spirulina (Arthrospira platensis) Dietary Supplements by DI-FT-ICR and Evaluation of their Antioxidant Potential by Pre-Column DPPH-UHPLC Assay
Source: Molecules. 2018 May 11;23(5):1152. doi: 10.3390/molecules23051152 (PMC6099715; doi:10.3390/molecules23051152)
Supplement: Supplementary file 1 [file molecules-23-01152-s001.pdf]

SUPPLEMENTARY MATERIAL FOR THE MANUSCRIPT

Fast profiling of natural pigments in different Spirulina (*Arthrospira Platensis*) dietary supplements by DI-FT-ICR and evaluation of their antioxidant potential by pre-column DPPH-UHPLC assay.

Eduardo Sommella[1], Giulio Maria Conte[1,2], Emanuela Salviati[1,2], Giacomo Pepe[1], Alessia Bertamino[1], Carmine Ostacolo[3], Francesca Sansone[1], Francesco Del Prete[4], Rita Patrizia Aquino[1], Pietro Campiglia[1]

[1] Department of Pharmacy, University of Salerno, Via Giovanni Paolo II 132, I-84084 Fisciano, SA, Italy

[2] PhD Program in Drug Discovery and Development, University of Salerno, Via Giovanni Paolo II 132, I-84084, Fisciano, SA, Italy

[3] Department of Pharmacy, University of Naples Federico II, Via D. Montesano 49, I-80131 Napoli, Italy

[4] European Biomedical Research Institute of Salerno, Via De Renzi 50, I-84125 Salerno, Italy

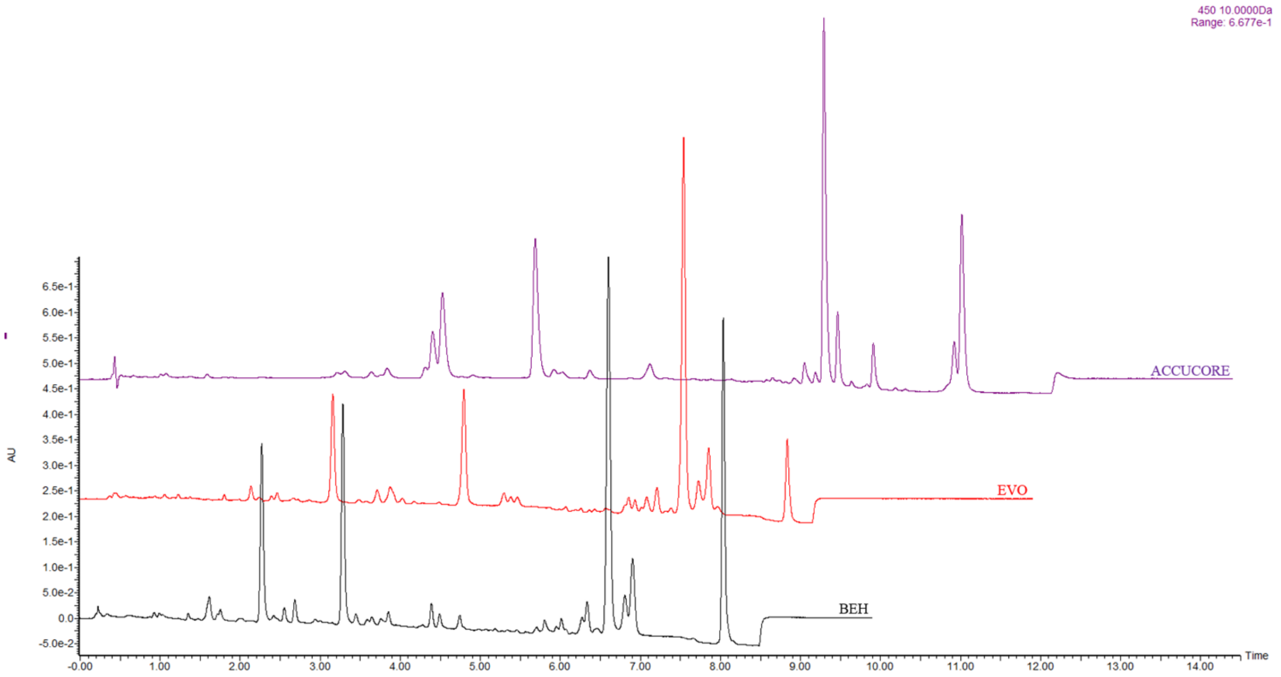

Figure S1: Comparison of UHPLC separation of Spirulina extract with three different columns: **(black)**: BEH C18 50 × 2.1 mm, 1.7 µm, **(red):** EVO C18 100 × 2.1 mm, 1.7 µm, **(purple):** C30 100 × 2.1 mm, 2.6 µm

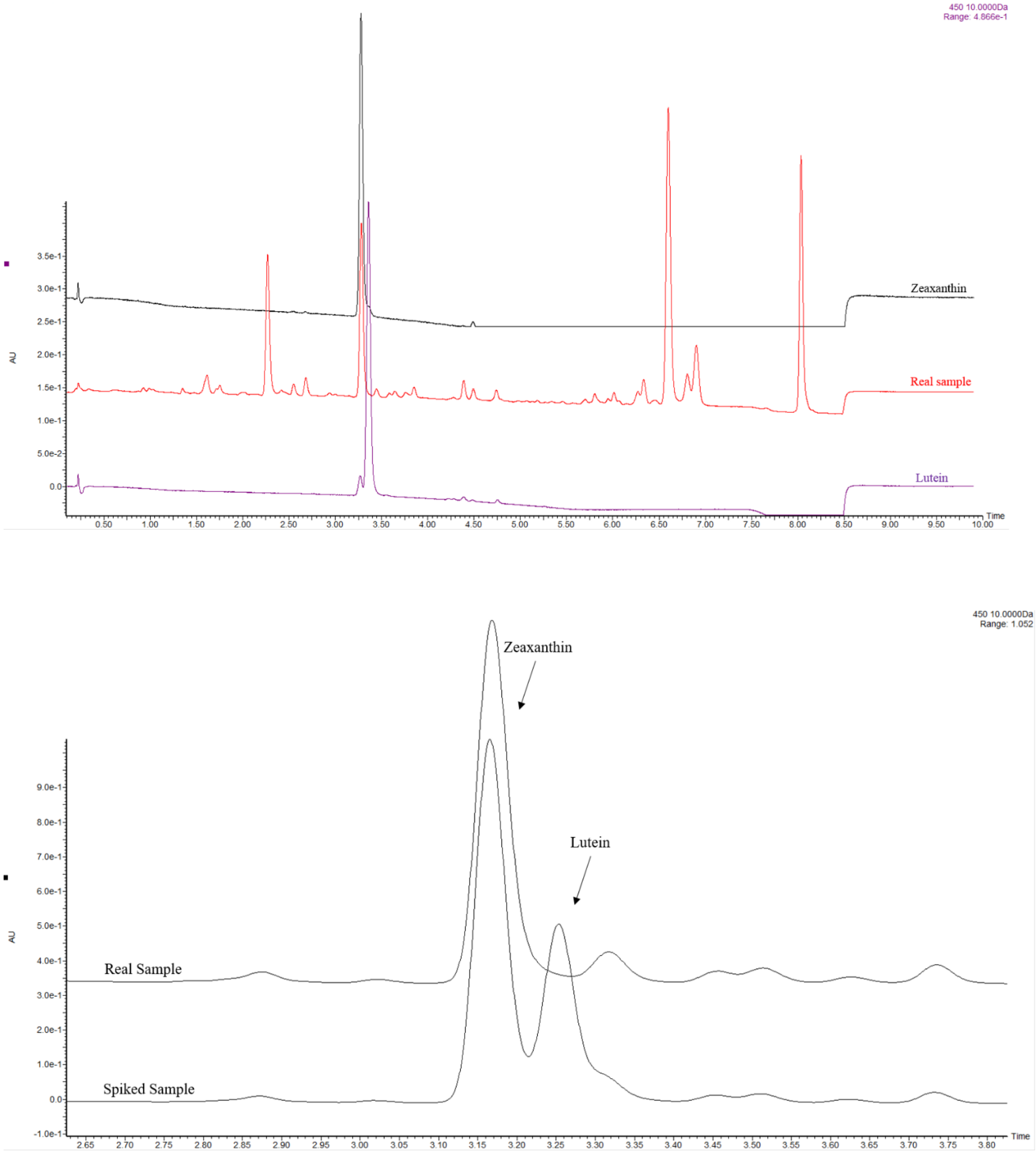

Figure S2: Comparison of real sample and standards of lutein and zeaxanthin

		INTRADAY		INTERDAY	
**Peak**	**Compounds**	***CV% Area***	***CV% Tr***	***CV% Area***	***CV% Tr***
**1**	***Diadinoxanthin***	0.007	0.009	0.150	0.015
**2**	***Canthaxanthin***	0.035	0.005	0.124	0.022
**3**	***Diatoxanthin***	0.011	0.004	0.045	0.022
**4**	***Antheraxanthin***	0.007	0.004	0.155	0.028
**5**	***Zeaxanthin***	0.002	0.003	0.068	0.027
**6**	***Echinone***	0.014	0.002	0.047	0.024
**7**	***Chlorophyll b***	0.011	0.002	0.315	0.026
**8**	***Hydroxychlorophyll a***	0.008	0.001	0.361	0.026
**9**	***Chlorophyll a***	0.004	0.002	0.006	0.031
**10**	***Chlorophyll a isomer***	0.006	0.001	0.051	0.034
**11**	***Beta carotene***	0.006	0.001	0.019	0.019

Table S1: Repeatability values for the UHPLC-PDA method

		Dietary supplement powder		*Lab made powder*		*Dietary supplement tablet*	
**Peak**	**Compounds**	***LOD µg/g***	***LOQ µg/g***	***LOD µg/g***	***LOQ µg/g***	***LOD µg/g***	***LOQ µg/g***
**1**	***Diadinoxanthin***	2.96	9.85	5.33	17.77	1.51	5.02
**2**	***Canthaxanthin***	1.14	3.81	4.52	15.07	0.75	2.49
**3**	***Diatoxanthin***	8.05	26.84	42.57	141.90	12.40	41.33
**4**	***Antheraxanthin***	0.58	1.92	6.06	20.22	4.65	15.49
**5**	***Zeaxanthin***	11.03	36.76	34.76	115.87	8.53	28.45
**6**	***Echinone***	5.47	18.22	2.22	7.40	5.38	17.93
**7**	***Beta carotene***	44.72	149.08	10.75	35.85	15.23	50.77

Table S2: LOD and LOQ values UHPLC-PDA method
